# Emergence of Southern Rice Black-Streaked Dwarf Virus in the Centuries-Old Chinese Yuanyang Agrosystem of Rice Landraces

**DOI:** 10.3390/v11110985

**Published:** 2019-10-25

**Authors:** Pascal Alonso, Pierre Gladieux, Oumaima Moubset, Pei-Jung Shih, Pierre Mournet, Julien Frouin, Laurence Blondin, Romain Ferdinand, Emmanuel Fernandez, Charlotte Julian, Denis Filloux, Henry Adreit, Elisabeth Fournier, Aurélie Ducasse, Vladimir Grosbois, Jean-Benoit Morel, Huichuan Huang, Baihui Jin, Xiahong He, Darren P. Martin, Christian Vernière, Philippe Roumagnac

**Affiliations:** 1 CIRAD, BGPI, 34398 Montpellier, France; pascal.alonso@cirad.fr (P.A.); oumaima.moubset@etu.umontpellier.fr (O.M.); y199404_20@yahoo.com.tw (P.-J.S.); laurence.blondin@cirad.fr (L.B.); romain.ferdinand@cirad.fr (R.F.); emmanuel.fernandez@cirad.fr (E.F.); charlotte.julian@cirad.fr (C.J.); denis.filloux@cirad.fr (D.F.); henri.adreit@cirad.fr (H.A.); christian.verniere@cirad.fr (C.V.); 2 BGPI, INRA, CIRAD, SupAgro, Univ Montpellier, 34398 Montpellier, France; pierre.gladieux@inra.fr (P.G.); elisabeth.fournier@inra.fr (E.F.); aurelie.ducasse@inra.fr (A.D.); jean-benoit.morel@cirad.fr (J.-B.M.); 3 INRA, BGPI, 34398 Montpellier, France; 4 Department of Plant Pathology, National Chung Hsing University, Taichung 402, Taiwan; 5 CIRAD, UMR AGAP, 34398 Montpellier, France; pierre.mournet@cirad.fr (P.M.); julien.frouin@cirad.fr (J.F.); 6 AGAP, Univ Montpellier, CIRAD, INRA, Montpellier SupAgro, 34398 Montpellier, France; 7 CIRAD, UMR ASTRE, 34398 Montpellier, France; vladimir.grosbois@cirad.fr; 8 State Key Laboratory for Conservation and Utilization of Bio-Resources in Yunnan, Yunnan Agricultural University, Kunming 650201, China; absklhhc@gmail.com (H.H.); hexiahong@hotmail.com (X.H.); 9 Southwest Forestry University, Kunming 650224, China; 10 Computational Biology Group, Institute of Infectious Diseases and Molecular Medicine, University of Cape Town, Cape Town 4579, South Africa; darrenpatrickmartin@gmail.com

**Keywords:** Southern rice black-streaked dwarf virus, rice, Honghe Hani rice terraces system, host genotyping, virus prevalence, virus emergence

## Abstract

Southern rice black-streaked dwarf virus (SRBSDV), which causes severe disease symptoms in rice (*Oriza sativa* L.) has been emerging in the last decade throughout northern Vietnam, southern Japan and southern, central and eastern China. Here we attempt to quantify the prevalence of SRBSDV in the Honghe Hani rice terraces system (HHRTS)—a Chinese 1300-year-old traditional rice production system. We first confirm that genetically diverse rice varieties are still being cultivated in the HHRTS and categorize these varieties into three main genetic clusters, including the modern hybrid varieties group (MH), the Hongyang improved modern variety group (HY) and the traditional *indica* landraces group (TIL). We also show over a 2-year period that SRBSDV remains prevalent in the HHRTS (20.1% prevalence) and that both the TIL (17.9% prevalence) and the MH varieties (5.1% prevalence) were less affected by SRBSDV than were the HY varieties (30.2% prevalence). Collectively we suggest that SRBSDV isolates are freely moving within the HHRTS and that TIL, HY and MH rice genetic clusters are not being preferentially infected by particular SRBSDV lineages. Given that SRBSDV can cause 30–50% rice yield losses, our study emphasizes both the need to better monitor the disease in the HHRTS, and the need to start considering ways to reduce its burden on rice production.

## 1. Introduction

Southern rice black-streaked dwarf virus (SRBSDV) causes the emergent rice black-streaked dwarf disease that has for the last decade been threatening rice-growing areas of northern Vietnam, southern Japan and southern, central and eastern China [1,2,3,4]. While the first report of black-streaked dwarf disease was in 2001 from a Chinese rice field in Guangdong, SRBSDV was only shown to be a novel fijivirus in 2008 [5,6]. Symptoms of SRBSDV infections include severe stunting, darkening of leaves, and white waxy or black-streaked swellings along stem veins that can lead to 30–50% rice yield losses [1,2]. The virus is transmitted by the white-backed planthopper *Sogatella furcifera*, but is not seed transmissible [2]. Besides rice, SRBSDV is able to infect several other species, including maize, sorghum and various uncultivated grasses [5,6]. The genome of SRBSDV is composed of ten linear dsRNA segments between 1.8 and 4.5 kb in length which encode twelve proteins [7]. Observed SRBSDV genetic diversity is low: for example, all characterized segment 8 sequences of Chinese SRBSDV isolates share more than 93.9% nucleotide sequence identity [8]. This low diversity has been interpreted as indirect evidence that SRBSDV has only recently emerged.

While SRBSDV is now known to be present in all provinces of Southern China, its prevalence and geographical distribution within each of these provinces remains unknown. Additionally, given that the prevalence of other plant viruses has been found to be higher in cultivated areas than in natural settings [9], it would be interesting to determine whether the relative prevalence of SRBSDV is lower in rice agronomic systems that use mixtures of landraces in comparison to those that use genetically-uniform modern varieties. For example, the prevalence and distribution of SRBSDV in the Honghe Hani rice terraces system (HHRTS) in the Yunnan province—a 1300-year-old traditional rice production system [10]—has never been precisely studied.

The HHRTS was developed over centuries by the Hani people and, amongst other peculiarities, is characterized by the cultivation of a remarkably diverse variety of locally bred rice landraces [10]. In 2008, each village in this mountainous region grew complex heterogeneous mosaics of rice genotypes drawn from at least 47 diverse rice landraces [10]. The Hani people have created stretched terraces that cascade down hillside slopes from 800 to 2000 m above sea level. Each terrace is composed of thin stretched fields within which only one rice landrace is grown. The diversity of landraces used in the HHRTS has for centuries apparently minimized the impacts of both biotic and abiotic stressors on rice production in this region [10]. For example, the long term cultivation of genetically diverse HHRTS landraces may have led today to the situation where two different lineages of the rice blast fungus, *Pyricularia oryzae,* are each locally adapted to either the *indica* or the *japonica* rice landraces that are grown in the terraces [11]. This specialization of different *P. oryzae* populations may limit the spread of this pathogen in the HHRTS, which, by extension, has reduced the impact of rice blast on rice yields in the terraces [11]. Given the relatively light burden of bacterial and fungal diseases affecting HHRTS landraces, in 2010, the Food and Agriculture Organization (FAO) designated the HHRTS as a “Globally Important Agricultural Heritage System” and, in 2013, the United Nations Educational, Scientific, and Cultural Organization (UNESCO) listed it as a World Cultural Heritage site.

However, the social, economic and environmental transformations that are presently occurring in the HHRTS are threatening the existence of this unique, highly successful, sustainable and durable agrosystem [10]. Specifically, chemical fertilizers are being increasingly used and genetically divergent landraces are being gradually replaced by high-yielding modern hybrid varieties [12]. A recent survey involving interviews with HHRTS farmers has revealed that those farmers who have attempted to incorporate modern fertilizers and hybrid varieties to achieve higher yields, have noticed a clear increase in the prevalence and severity of rice diseases [13].

Here we attempt to both quantify the prevalence of SRBSDV in the HHRTS and determine whether this emergent virus has a greater impact on modern high yielding rice varieties than it does on traditional HHRTS landraces. We first confirm that genetically diverse rice varieties are still being cultivated in the HHRTS and categorize these varieties into three main genetic clusters: the traditional *indica* landraces group (TIL); the Hongyang group (HY) that contains improved modern varieties sharing recent ancestry with the TIL varieties; and the modern hybrid varieties group (MH) that includes commercial varieties usually grown outside the HHRTS. We show over a period of two years that SRBSDV remains highly prevalent in the HHRTS (20.1% prevalence) and that both the TIL (17.9% prevalence) and the MH varieties (5.1% prevalence) were less affected by SRBSDV than were the HY varieties (30.2% prevalence)

## 2. Material and Methods

### 2.1. Study Area

While the upper reaches of the Malizhai River Basin in the vicinity of Xinjie town in the Honghe Hani Yi Autonomous Prefecture (Yunnan province, China) is representative of the cultural landscape of the Hani rice terraces [10], we focused our study on villages located in this river basin. The Hani people are perpetuating land-management practices related to the cultivation of irrigated rice terraces that include profound and long-standing understanding of environmental conditions such as landforms, soil conditions, vegetation types, and hydrology. However, rice landraces are being gradually replaced by high-yielding modern hybrid varieties in the Malizhai River Basin [12] and part of Hani farmers from the Malizhai River Basin are adopting mixed landrace/modern variety systems [13]. Specifically, Hani farmers are transplanting landrace and modern varieties seedlings into the fields after 60- and 40-days germination in the nursery plot, respectively. Neither chemical fertilizer nor fungicide and pesticide are usually applied in the field during the whole growth stages of the plants.

### 2.2. Plant Sampling

Seven villages from the upper Malizhai River Basin were prospected, including one village (Malizhai altitude of 1570–1608 m) in 2016 and six villages (Xiaoshuijing, 1781 m alt.; Tuguozhai, 1778–1793 m alt.; Shuibulong, 1647–1666 m alt.; Huangcaolin, 1732–1747 m alt.; Gingko, 1629 m alt.; and Dayutang, 1769–1805 m alt.) in 2018 (Figure S1). Both traditional landraces and modern rice varieties were cultivated in these seven villages. In each village, approximately equal numbers of leaf samples from modern varieties and traditional landraces were collected by farmers and government technicians without regard for the presence of disease symptoms. Seven to ten plants were collected in each of the sampled rice fields (Table 1). Collectively, these accounted for 186 samples collected in 2016 and 173 samples collected in 2018 (Table 1). All of these plants were assumed to have grown under the same weather, water, soil and nutrient conditions.

### 2.3. Genotyping-by-Sequencing of Rice Landraces

Total genomic DNA extractions were performed from 30 mg of dried individual leaf samples. Plant cell were lysed using the mixed alkyltrimethylammonium bromide (MATAB) buffer [14] at 72 °C for 30 min. DNA was then extracted using an automated method on a Biomek FXP instrument (Beckman Coulter, CA, USA) [15] that uses the NucleoMag Plant Kit (Macherey–Nagel, Germany). DNA concentrations were quantified with Hoescht dye and a Fluoroskan Ascent FL fluorometer (Thermo Fisher Scientific, Waltham, MA, USA). Genomic DNA quality was further checked using agarose gel electrophoresis. Two genomic libraries (one library containing the 186 samples collected in 2016 and the other the 173 samples from 2018) were prepared as previously described [16] using *Ape*KI restriction enzyme digestion (New England Biolabs, Hitchin, UK) from 100 ng of DNA per sample. A ligation reaction was then carried out using T4 DNA ligase (New England Biolabs, Hitchin, UK) and barcoded adapters at 22 °C for 30 min [16], then ligase was inactivated by heating at 65 °C for 20 min. Barcoded samples were then pooled and PCR-amplified. PCR reactions were carried out in a 50 μL volume consisting of 5 µL of pooled and barcoded samples, 2 μL of each primer (10 µmol/μL), 25 μL of NEB 2X *Taq* Master Mix (NEB#M0270S; New England Biolabs, Hitchin, UK) and RNAse-free water to 50 µL. The PCR program was as follows: 72 °C for 5 min, 98 °C for 30 s, 18 cycles at 98 °C for 10 s, 65 °C for 30 s and 72 °C for 30 s with a final 72 °C extension for 5 min. The PCR-amplified libraries were purified using the Wizard PCR prep DNA purification system from Promega (Madison, WI, USA) and verified with the Agilent D5000 ScreenTape instrument (Santa Clara, CA, USA). While the 2016-library was sequenced at the GeT-PlaGe platform at Toulouse (France) using a single lane on an Illumina HiSeq system (2 × 150 bp sequencing), the 2018 library was sequenced by Genewiz (Leipzig, Germany) using a single lane on an Illumina HiSeq system (2 × 150 bp sequencing). The raw sequence read data from these two Illumina HiSeq runs are available from the NCBI Sequence Read Archive, under the study accession number: PRJNA573048

### 2.4. Analysis of Rice Genotyping-by-Sequencing Data

Read processing, read mapping and single nucleotide polymorphism (SNP) calling were carried out using bioinformatic pipelines designed using Toggle (https://github.com/SouthGreenPlatform/TOGGLE). Reads were aligned to the Nipponbare rice reference genome (https://plants.ensembl.org/Oryza_sativa/Info/Index) using Burrows Wheeler alignment (BWA) [17], and SNPs were called using UnifiedGenotyper in GATK v. 3.8 [18]. Only positions in coding sequences, with DP > 3 and MQ > 20, and with less than 50% missing data were retained. The final dataset used for genotyping consisted of 12,112 SNPs. Accessions Plot-22-P3 and YYT10-A were removed from the data as proportions of missing data in these exceeded 95%.

We constructed a neighbor-network using SplitsTree 4.13 [19], to visualize evolutionary relationships between the *indica* rice genotypes while taking the possibility of recombination or incomplete lineage sorting into account. We also used the program sNMF (http://membres-timc.imag.fr/Olivier.Francois/snmf/index.htm) to infer population subdivision by partitioning genotypes into K ancestral populations and estimating individual ancestry coefficients in the K populations. We ran sNMF for K ranging from 1 to 20, and for each K value 10 replications were performed. We used the Greedy algorithm in CLUMPP 1.1.2 (http://web.stanford.edu/group/rosenberglab/clumpp.html) to identify runs belonging to the same mode (i.e., representing the same clustering solution) and we randomly selected one representative of the major mode for graphical representation as a stacked barplot using the Matplotlib package in Python.

### 2.5. RNA Extraction and Detection of SRBSDV by Real-Time Quantitative PCR

Virions were initially concentrated from approximatively 100 mg of rice leaf tissue using the virion-associated nucleic acids (VANA) protocol as previously described [20]. Total RNA of the virion preparation was extracted with the QIAGEN^®^ RNeasy Plant Mini Kit (Qiagen, Valencia, CA, USA) as described by the manufacturer. Three-hundred and fifty-nine plant sample RNAs (186 from 2016 and 173 from 2018) were tested for the presence of SRBSDV using quantitative real-time PCR (qPCR). The previously designed primers [21], s9-1-F (5′-AACGACCAACCAACAAGA-3′) and s9-1-R (5′-GTTCCATCAATGAGGTAGTTC-3′) were used. The reverse transcription and the qPCR reaction were performed using the QIAGEN^®^ OneStep RT-PCR Kit and the Invitrogen™ Quant-iT™ PicoGreen™ dsDNA reagent on a Stratagene Mx3000P instrument. The 25 μL RT-qPCR reaction mix consisted of 2 μL of eluted RNA, 13 μL of RNAse-free water, 5 μL of QIAGEN OneStep RT-PCR buffer (5×), 1 μL of dNTP mix (10 mM), 1.25 μL of each primer (10 μM), 1 µL of PicoGreen 1× and 1 μL of QIAGEN OneStep RT-PCR enzyme mix. The RT-qPCR program was as follows: 50 °C for 30 min, 95 °C for 15 min, 40 cycles at 94 °C for 1 min, 58 °C for 1 min and 72 °C for 1 min with a final 72 °C extension for 10 min followed by a melt curve step (from 55 °C, gradually increasing 0.5 °C/s to 95 °C, with acquisition data every 1 s).

### 2.6. Reverse-Transcription PCR, Partial SRBSDV Segment 8 Sequencing and Sequence Analysis

A fragment of SRBSDV segment 8 encoding a putative core structural protein [21] was amplified from 26 SRBSDV isolates sampled from the HHRTS (including 20 isolates from 2016 and six isolates from 2018) by RT-PCR using primers SRBSDV_S8_F3 (5′-GGG TTG ATT CCT TTG GT-3′) and SRBSDV_S8_R3 (5′-ACG GGA TTG TCT CCT TTG-3′) [8]. The RT-PCR reaction was performed as described above without adding PicoGreen reagent. The PCR product was analyzed by electrophoresis on a 1.0% agarose gel in TAE 1X buffer stained with ethidium bromide and visualized under UV light. The SRBSDV amplification product had an expected size of 776 bp. Amplicons were directly sequenced using automated Sanger sequencing (Genewiz, South Plainfield, NJ, USA). SRBSDV segment 8 fragments were further aligned using MUSCLE [22] as implemented in MEGA with default settings [23] and manually trimmed (down to 624 nt in length). Pairwise identity analyses of partial segment 8 sequences were carried using SDT v1.2 [24].

### 2.7. Phylogenetic Analysis

Twenty-six SRBSDV segment 8 fragments (624 nt in length) and all 29 of the SRBSDV segment 8 sequences that were publicly available in June 2019 were aligned using MUSCLE [22] as implemented in MEGA (with default settings). A maximum likelihood phylogenetic tree was constructed using PhyML3 [25] with the T92 nucleotide substitution model (selected as best fit by MEGA) and 1000 bootstrap replicates were used to test the support of branches.

### 2.8. Statistical Analyses

We applied a generalized linear model (GLM) implemented in *R Base* (http://www.r-project.org) to analyze proportions of SRBSDV-infected plants (number of diseased plants per plot / total number of collected plants in each plot) in 2016 and 2018. Sampling plots were split into three rice variety groups: the TIL group, the HY improved modern variety group and the MH variety group (Table 1). As variation in the number of SRBSDV-infected plants given the number of plants collected was greater than that expected under standard binomial models, a phenomenon referred to as overdispersion, the beta-binomial model was further fitted to our data. The effect of rice variety group and the year of sampling were tested using two GLM models: (1) a model where the two explanatory variables were considered as having additive effects (variety group + year of sampling); and (2) a model additionally including an interaction between the two explanatory variables (variety group + year of sampling + variety group: year of sampling). Tukey’s multiple comparison test (function Tukey HSD in *R*) was further used to test for pairwise differences in SRBSDV prevalence among variety groups.

## 3. Results and Discussion 

### 3.1. Highly Genetically Diverse TIL, HY and MH Varieties Are Cultivated in the HHRTS

**Figure 1:**
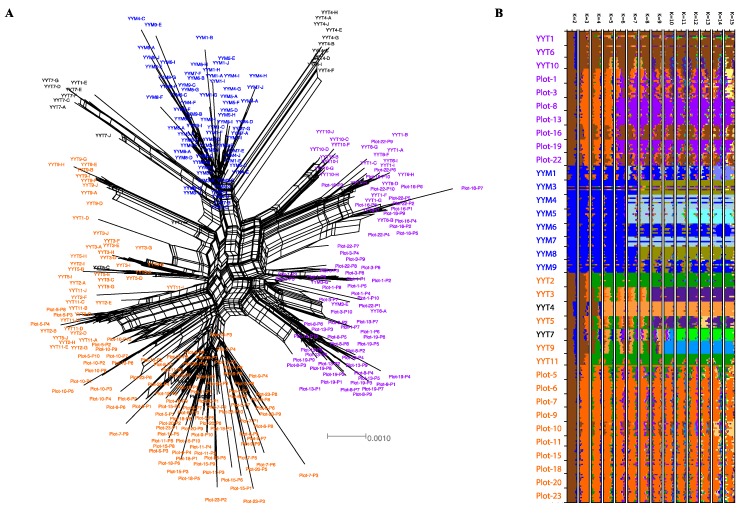
(**A**) Neighbor-Net split decomposition network indicating the relationships between rice accessions based on 12,112 analyzed SNPs. Plant samples assigned to the HY, MH and TIL *indica* variety groups are labelled in purple, blue and orange, respectively. Plant samples that were not assigned to one of the three variety groups (“outlier varieties”) are labelled in black. Characters A to J or P1 to P10 (e.g., YYT5-J or Plot-23-P2) refer to individual plants that were collected from each rice field (7–10 plants were collected in each field, Table 1) (**B**) Ancestry proportions within accessions for sNMF models [26] from K = 2 to K = 15 ancestral populations. Each horizontal bar represents the proportion of ancestry within a single accession, with colors corresponding to ancestral populations.

Rice genotyping-by-sequencing confirmed that the HHRTS hosts a large diversity of rice varieties, including traditional *indica* or *japonica* local rice subspecies and modern *indica* varieties that are widely grown in other regions of China and elsewhere in South-East Asia (Table 1). The genotyping also confirmed that only one rice variety was grown in each of the small rice fields that were sampled in this study (Figure 1A and Figure S1). Specifically, we show that, besides TILs, two groups of modern *indica* varieties were being grown in the HHRTS in 2016 and 2018: (1) the HY variety group; and (2) the MH variety group (Figure 1A). The admixture analysis revealed that the HY varieties that have been in use in the HHRTS since 2010 [13] share recent ancestry with the TIL varieties (K = 2 to K = 5 models in Figure 1B).

Accordingly, the branches connecting the HY and TIL varieties in a split decomposition network constructed from 12,112 SNPs were more reticulated than the branches connecting HY or TIL varieties to the MH varieties, indicating conflicting phylogenetic signals caused by recent shared ancestry between HY and TIL, but not between MH and HY/TIL (Figure 1A). This split decomposition network structure suggests that the MH varieties that are commercially grown outside the HHRTS have a narrow genetic base and have likely been bred from rice lineages which do not share any recent ancestries with either TIL or HY local varieties. The lack of shared ancestry between MH and TIL/HY in our admixture analysis also supports this hypothesis (Figure 1B). Finally, two varieties (YYT4 and YYT7, Table 1) that were considered to be traditional landraces by the farmers, did not cluster phylogenetically with the TIL, HY or MH groups (Figure 1A) and are therefore hereafter referred to as “outlier” varieties. Collectively these results demonstrate the presence of strong rice population structure within the HHRTS with a group of *japonica* varieties, three large groups of *indica* varieties and two other *indica* varieties, reflecting divergent breeding histories.

### 3.2. SRBSDV Prevalence and Distribution

A total of 359 rice leaf samples from the HHRTS (186 from 2016 and 173 from 2018) were tested for the presence of SRBSDV, 60 of which (20.1%) tested positive by qPCR (Table 1). These consisted of 23 samples collected in 2016 (12.4% of the 2016 samples, Table 1) and 37 collected in 2018 (21.4% of the 2018 samples, Table 1). SRBSDV was detected in every village where plants were sampled, and virus prevalence ranged from 7.1% in Shuibulong village to 50% in Dayutang village (Table 2). These results indicate that, as is the case elsewhere in South China and northern Vietnam [1,2,3,4], SRBSDV is highly prevalent in the HHRTS. This brings into question the assumption that, in general, viruses causing rice diseases have low prevalence in the HHRTS [10]. Although this indicates that SRBSDV may at present be a major biotic constraint on rice production in the HHRTS, it is plausible that, given enough time, the HHRTS farmers will eventually select additional SRBSDV-tolerant/resistant varieties for use in the terraces.

While long-term monitoring of SRBSDV prevalence in the HHRTS will be needed to determine how successfully such selective breeding efforts will be, a reasonable first step towards controlling the virus in the short term will be to determine which of the HY, MH and TIL varieties are most susceptible to infection by SRBSDV. We found that among the rice plants that were detectably infected with the virus, the HY varieties have the highest SRBSDV prevalence (30.2%) with infected HY plants being found in 8/10 sampled HY fields (Table 1 and Table 2). Both the TIL (17.9% prevalence occurring in 10/15 TIL fields) and MH varieties (5.1% prevalence in 3/8 MH fields) were less affected by SRBSDV than were the HY varieties (Table 1 and Table 2). Furthermore, none of the 20 samples from the two “outlier” *indica* landraces, which are phylogenetically most closely related to the MH varieties (Figure 1A), were detectably infected by SRBSDV (Table 1). Finally, the *japonica* plant samples were rarely affected by SRBSDV (5% prevalence in 1/2 *japonica* fields, Table 1).

Given the observed differences in the detected prevalence of SRBSDV in the different rice variety groups, we tested whether the variety groups (HY, TIL and MH) or the year of sampling (2016, 2018) displayed statistically significant differences in virus prevalence using a GLM-based approach. The model including the interaction between variety group and year of sampling revealed that there was no significant interaction between these explanatory variables (*p* = 0.067). Consequently, we selected the additive model that indicated that the SRBSDV prevalence observed for the different variety groups were significantly different (*p* = 0.012). In addition, pairwise comparisons using the Tukey HSD test indicated that there was significantly higher prevalence of the virus in the HY varieties than in the MH varieties (*p* = 0.024) but no significant difference in prevalence between the HY and TIL varieties (*p* = 0.241), or between the TIL and MH varieties (*p* = 0.160).

Interestingly, there was no significant difference in SRBSDV prevalence between 2016 and 2018 (*p* = 0.710). The sustained prevalence of the virus between 2016 and 2018 suggests that SRBSDV is possibly now endemic in the HHRTS system.

### 3.3. SRBSDV Genetic Diversity

The degree of genetic diversity within the amplified HHRTS SRBSDV genome segment 8 sequences was compared to that of 29 other publicly available SRBSDV segment 8 sequences collected since 2003 from several regions of China and Vietnam. The 26 fragments (624 bp long after alignment and trimming of the 5′- and 3′-ends of the sequences; GenBank accession numbers: MN244953- MN244978) from the HHRTS share between 98.2% and 100% pairwise identity with one another and between 94.1% and 100% pairwise identity with the 29 publicly available SRBSDV segment 8 sequences. Interestingly, all SRBSDV sequences from rice plants are genetically highly homogeneous (sharing between 98.2% and 100% pairwise identity with one another) whereas they share slightly lower identities with SRBSDV isolates sampled from maize (between 94.1% and 99% pairwise identity).

Overall, these degrees of sequence identity are relatively high, and are consistent with SRBSDV having emerged and spread to rice plants throughout Southern China and Northern Vietnam in the recent past. Specifically, the genetic distance between the two most divergent Chinese and Vietnamese rice isolates is only 0.018 nucleotide differences per site implying that the genetic distance of these most divergent isolates to their most recent common ancestor is approximately 0.018/2 = 0.009 nucleotide differences per site. If SRBSDV did indeed emerge in approximately 2000 this would suggest a nucleotide substitution rate in the order of approximately 5 × 10^−4^ substitutions per site per year: a substitution rate which is in the middle of the range of those estimated previously for other double stranded RNA viruses [27,28,29,30,31,32].

**Figure 2:**
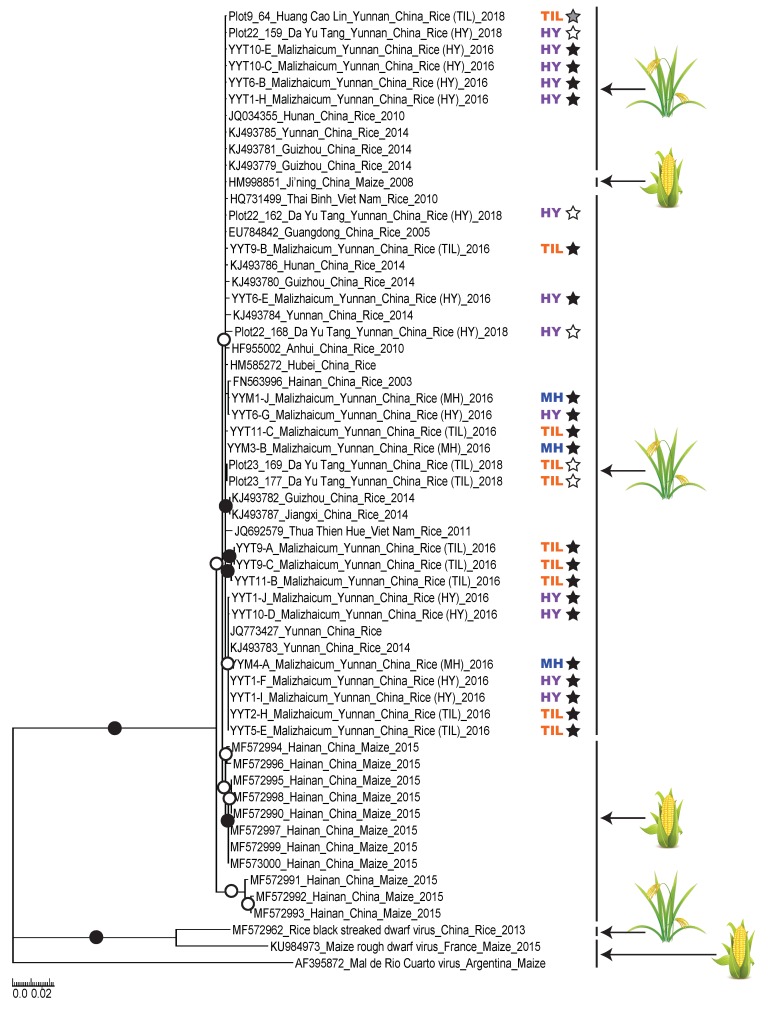
Maximum-likelihood phylogenetic trees of partial SRBSDV segment 8 sequences (624 nt in length). Branches with less than 50% bootstrap support were collapsed. Branches associated with a black dot have bootstrap supports above 90% whereas those with white dots have bootstrap supports above 70%. Variety group of the samples, the village where the plant sample was collected and the host plant are depicted on the right of the phylogenetic tree. Variety groups: traditional *indica* landraces group (TIL); Hongyang improved modern variety group (HY) and modern hybrid variety group (MH). Villages: grey star, Huangcaolin village; black star, Malizhai village and white star, Dayutang village.

A maximum likelihood phylogenetic tree containing all 55 of the available SRBSDV partial segment 8 sequences together with homologous genome fragments of three other closely related fijivirus species (included for rooting purposes) indicated that SRBSDV isolates from the Chinese Hainan region and the SRBSDV clade (including all isolates from all other regions) are sister groups (Figure 2).

It is noteworthy that, while all of these isolates from the Hainan region were isolated from maize, all but one of the other isolates from the SRBSDV clade were isolated from rice plants collected from China and Vietnam (Figure 2). These results are consistent with SRBSDV having originated in maize —possibly in the Hainan region—prior to it spreading into the rice fields of South China and Northern Vietnam with the recurrent annual migrations of viruliferous white back planthopper *Sogatella furcifera*. However, properly testing this hypothesis will require both the genomic sequence characterization and analysis of many more SRBSDV isolates from rice and maize plants throughout the entire current geographical range of the virus, and an accurate estimate of the SRBSDV nucleotide substitution rate. With this data in hand it should be possible to determine the most likely time when, and place where, the SRBSDV isolates that are responsible for rice black-streaked dwarf disease first arose.

Although all of the SRBSDV isolates from the HHRTS clustered within the SRBSDV “rice clade” (Figure 2) there was no evidence to indicate that they may have all shared a more recent common ancestor with one another than with other SRBSDV isolates from China and Vietnam. This suggests that there have likely been multiple (and possibly ongoing) transmissions of SRBSDV into the HHRTS.

Also, within the HHRTS we were unable to find any phylogenetic evidence to support the hypothesis that particular rice variety groups (i.e., TIL, HY and MH) were being preferentially infected by particular SRBSDV lineages. Rather, the SRBSDV isolates obtained from the three variety groups, or from different villages in different years, were interspersed throughout the maximum likelihood tree: a pattern indicating both the persistence of viruses between years, and frequent transmissions of viruses between the variety groups and between the villages (Figure 2). Collectively the phylogenetic evidence supports the hypotheses that SRBSDV isolates are freely moving within the HHRTS and that no obvious rice genotype-specific specialization of SRBSDV isolates has occurred.

### 3.4. The Emergence of SRBSDV and Long-Term HHRTS Sustainability

Contrary to past observations and predictions [10], we have revealed that a viral disease has emerged and become endemic in the HHRTS. Our study also revealed that the prevalence of the virus that causes this disease, SRBSDV, is significantly higher in the HY variety group than in the MH variety group. In addition, while not significant, the prevalence of SRBSDV in HY varieties is also higher than the prevalence in TIL varieties. This observation is consistent with a recent survey which found that farmers who since 2014 had shifted to using high-yielding HY varieties had perceived a subsequent clear increase in disease prevalence and severity [13]. Our results suggest that the rice black-streaked dwarf disease may have been responsible for this perception of “diseased plants” by the interviewed farmers.

Given that rice black-streaked dwarf disease can cause 30–50% rice yield losses [1,2], our study emphasizes both the need to better monitor the disease in the HHRTS, and the need to start considering ways to reduce its burden on rice production within the terraces. Our finding that different rice variety groups are differentially infected by SRBSDV is consistent with the notion that continuing with the traditional use of highly genetic diverse rice landraces in the HHRTS should reduce the overall impact of SRBSDV within the system. Specifically, the absence of detectable SRBSDV infections in several of the TIL, MH and HY varieties (Table 1) may indicate that the rice genetic resources for minimizing the impacts of SRBSDV on the HHRTS are already at hand. Continuing the HHRTS tradition of farmer-led selection of TIL and other varieties that are least affected by the virus may eventually protect the system from this virus.

A major challenge will be the maintenance of the secular HHRTS farm seed exchange organizations that rely on a variety of non-market mechanisms such as gifts, social reciprocity networks and shared infrastructure resources to enable the dissemination of TIL varieties [13]. In addition, our study shows that several MH and HY SRBSDV-tolerant rice varieties, could also be selected and used in the HHRTS. However, more studies are needed to assess the long-term susceptibility of these rice varieties, to monitor the changing impacts of rice black-streaked dwarf disease on yields in the HHRTS, and to better estimate the optimal proportions of modern vs. TIL varieties that should be deployed in the HHRTS to ensure both the maximization of rice yields and the continuing development of new TIL varieties within the terraces.

## Figures and Tables

**Table 1:** Genotyping of rice varieties from the Yuanyang rice terraces of China and screening of these varieties for the presence of Southern rice black-streaked dwarf virus using quantitative real-time PCR (qPCR) assays. HY: Hongyang improved modern variety group, MH: modern hybrid variety group, SRBSDV: Southern rice black-streaked dwarf virus, TIL: traditional *indica* landraces group and Na: not assigned.

Sampling Field	Variety Group	Year	Rice Variety Name	Village	Number of Samples	SRBSDV Positive Samples	SRBSDV Negative Samples
YYT1	HY	2016	Chepugu	Malizhai	9	5	4
YYT6	HY	2016	Chepugu	Malizhai	9	4	5
YYT10	HY	2016	Jinpinggu	Malizhai	10	4	6
Plot_1	HY	2018	Hongyang	Gingko	10	1	9
Plot_3	HY	2018	Hongyang	Gingko	10	3	7
Plot_8	HY	2018	Hongyang	Huangcaolin	9	2	7
Plot_13	HY	2018	Hongyang	Xiaoshuijing	10	0	10
Plot_16	HY	2018	Hongyang	Tuguozhai	10	4	6
Plot_19	HY	2018	Hongyang	Shuibulong	9	0	9
Plot_22	HY	2018	Hongyang	Dayutang	10	6	4
YYM1	MH	2016	Mingliangyou 527	Malizhai	10	1	9
YYM3	MH	2016	Mingliangyou 528	Malizhai	8	1	7
YYM4	MH	2016	Hefeng 177	Malizhai	10	2	8
YYM5	MH	2016	Zhongyou 177	Malizhai	10	0	10
YYM6	MH	2016	Liangyou 2186	Malizhai	10	0	10
YYM7	MH	2016	Guofeng 1	Malizhai	10	0	10
YYM8	MH	2016	Liangyou 725	Malizhai	10	0	10
YYM9	MH	2016	Liangyou 2161	Malizhai	10	0	10
YYT2	TIL	2016	Lubaigu	Malizhai	10	1	9
YYT3	TIL	2016	Zaogu	Malizhai	10	0	10
YYT5	TIL	2016	Epugu	Malizhai	10	1	9
YYT9	TIL	2016	Nuogu	Malizhai	10	4	6
YYT11	TIL	2016	Luhonggu	Malizhai	10	0	10
Plot_5	TIL	2018	Acuce	Gingko	10	2	8
Plot_6	TIL	2018	Acuce	Gingko	10	3	7
Plot_7	TIL	2018	Acuce	Huangcaolin	9	1	8
Plot_9	TIL	2018	Acuce	Huangcaolin	10	5	5
Plot_10	TIL	2018	Acuce	Xiaoshuijing	10	0	10
Plot_11	TIL	2018	Acuce	Xiaoshuijing	7	3	4
Plot_15	TIL	2018	Acuce	Tuguozhai	10	0	10
Plot_18	TIL	2018	Acuce	Shuibulong	9	0	9
Plot_20	TIL	2018	Acuce	Shuibulong	10	2	8
Plot_23	TIL	2018	Acuce	Dayutang	10	4	6
YYT4	Na (outlier)	2016	Nuogu	Malizhai	10	0	10
YYT7	Na (outlier)	2016	Jianshuigu	Malizhai	10	0	10
YYT8	*japonica*	2016	Honglueduolu	Malizhai	10	0	10
Plot_12	*japonica*	2018	Unknown	Xiaoshuijing	10	1	9

**Table 2:** Screening of rice samples from the Yuanyang rice terraces of China for the presence of Southern rice black-streaked dwarf virus using qPCR assays. Rice varieties are grouped in rice variety groups (rows) or villages from which they were collected (columns). HY: Hongyang improved modern variety group, MH: modern hybrid variety group, TIL: traditional *indica* landraces group and Na: not assigned. SRBSDV prevalence is indicated within brackets and bold characters.

Year	2016	2018					
**Village**	**Malizhai** **(12.4%)**	**Xiaoshuijing** **(10.8%)**	**Tuguozhai** **(20%)**	**Shuibulong** **(7.1%)**	**Huangcaolin** **(28.6%)**	**Gingko** **(22.5%)**	**Dayutang** **(50%)**
**TIL** **(17.9%)**	6/50 **(12%)**	3/17 **(17.6%)**	0/10	2/19 **(10.5%)**	6/19 **(31.6%)**	5/20 **(25%)**	4/10 **(40%)**
**HY** **(30.2%)**	13/28 **(46.4%)**	0/10	4/10 **(40%)**	0/9	2/9 **(22.2%)**	4/20 **(20%)**	6/10 **(60%)**
**MH** **(5.1%)**	4/78 **(5.1%)**						
**Na (outlier)**	0/20						
***Japonica*** **(5.3%)**	0/10	1/10 **(11.1%)**					

## Supplementary Materials

The following are available online at https://www.mdpi.com/1999-4915/11/11/985/s1, Figure S1: (A) Map of Asia and the location of the Honghe Hani rice terraces system (HHRTS). (B) Map of the Malizhai River Basin from HHRTS and location of the seven villages where rice plants were collected in 2016 and 2018. (C) Map of the Malizhai village and location of the rice fields were collected in 2016.
