# Supplementary figures and images for: Emergence of Southern Rice Black-Streaked Dwarf Virus in the Centuries-Old Chinese Yuanyang Agrosystem of Rice Landraces
Source: Viruses. 2019 Oct 25;11(11):985. doi: 10.3390/v11110985 (PMC6893465; doi:10.3390/v11110985)

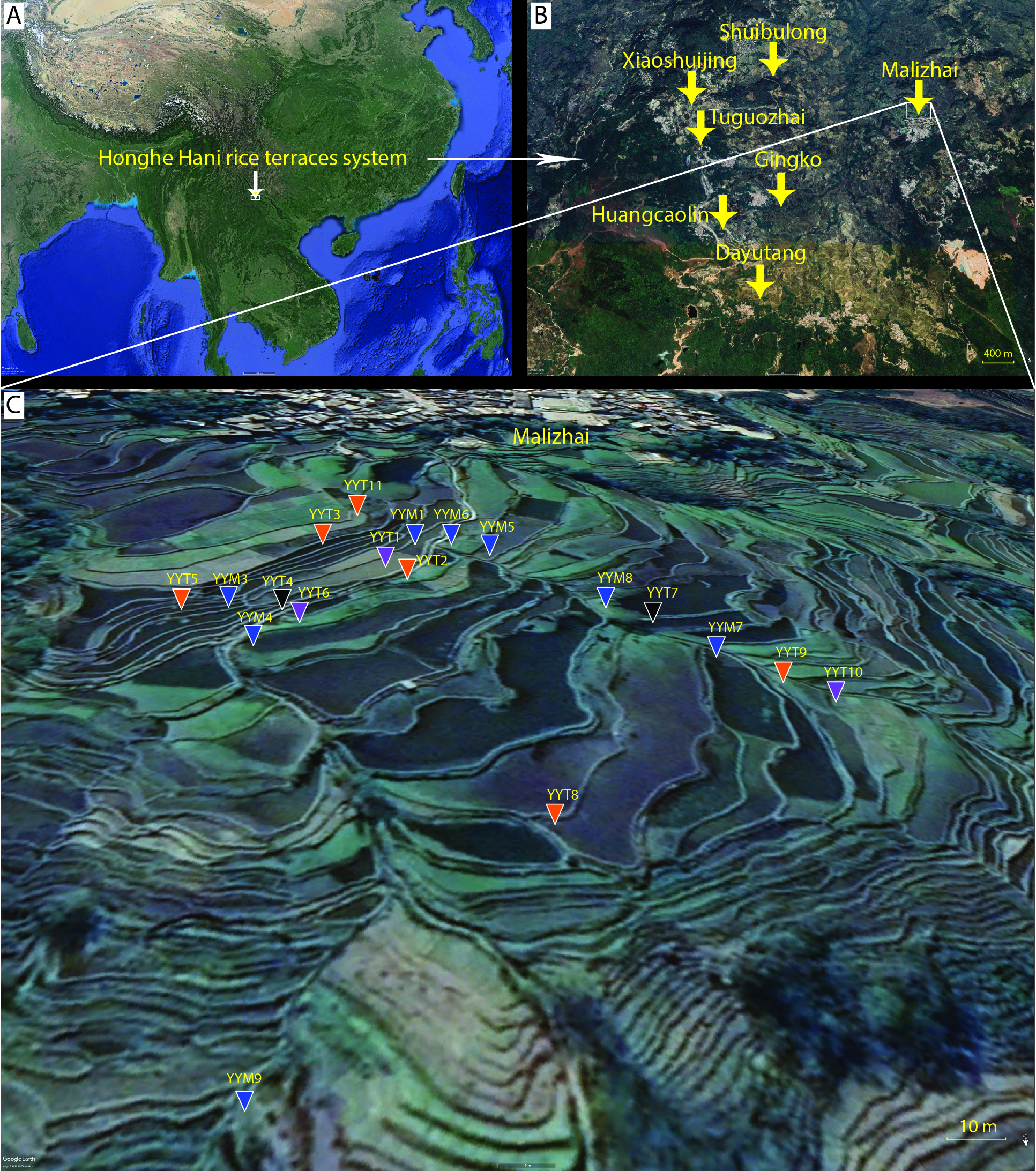

Supplement: Supplementary file 1 [file viruses-11-00985-s001.zip › viruses-612373-SI.jpg]
